# Gene assembly *via* one-pot chemical ligation of DNA promoted by DNA nanostructures†
†Electronic supplementary information (ESI) available: Experimental procedures, supplementary figures, sequencing data, oligonucleotides sequences. See DOI: 10.1039/c8cc00738a


**DOI:** 10.1039/c8cc00738a

**Published:** 2018-04-17

**Authors:** Ilenia Manuguerra, Stefano Croce, Afaf H. El-Sagheer, Abhichart Krissanaprasit, Tom Brown, Kurt V. Gothelf, Antonio Manetto

**Affiliations:** a Interdisciplinary Nanoscience Center (iNANO) , Gustav Wieds Vej 14 and Department of Chemistry , Aarhus University , Langelandsgade 140 , 8000 Aarhus , Denmark; b Center for Integrated Protein Science , Department of Chemistry , Ludwig-Maximilians-Universität (LMU) , Butenandtstrasse 5-13 , 81377 Munich , Germany; c Baseclick GmbH , Floriansbogen 2-4 , 82061 Neuried Munich , Germany; d Department of Chemistry , University of Oxford , Chemistry Research Laboratory , 12 Mansfield Road , Oxford , OX1 3TA , UK; e Chemistry Branch , Department of Science and Mathematics , Faculty of Petroleum and Mining Engineering , Suez University , 43721 Suez , Egypt; f Metabion GmbH , Semmelweisstrasse 3 , 82152 Planegg Munich , Germany . Email: a.manetto@metabion.com

## Abstract

Current gene synthesis methods are driven by enzymatic reactions. Here we report the one-pot synthesis of a chemically-ligated gene from 14 oligonucleotides. The chemical ligation benefits from the highly efficient click chemistry approach templated by DNA nanostructures, and produces modified DNA that is compatible with polymerase enzymes.

## Abstract 2

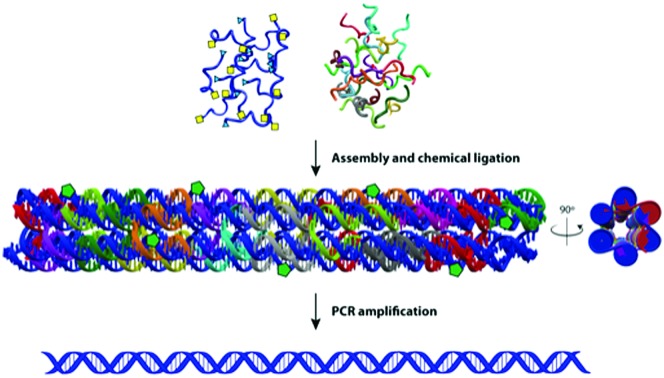
A gene was obtained from 14 oligonucleotides self-assembled and chemically ligated in a DNA nanostructure.

## 

The DNA nanotechnology and synthetic biology fields rely on synthetic oligonucleotides that are assembled to form nanostructures and artificial genetic systems. Synthesis of high quality oligonucleotides by solid phase synthesis^1^ depends among other factors on the sequence length, nucleotide composition and the purification system used. Yields exceeding 99% are not rare for each coupling step, although even the most efficient synthesis setup cannot reach 100% coupling efficiency. Therefore, the overall percentage yield of oligonucleotides strongly depends on their length. For instance, the synthesis of a 200-mer, where each cycle has an efficiency of incorporation of 99%, yields 13% of full-length product without taking in account further purification steps. Therefore, synthesis of oligonucleotides that are shorter than 100 nts is preferred in order to achieve reliable yields. Furthermore, the acidic reagents used for the de-tritylation step can lead to the formation of abasic sites and cleavage of the biopolymer, further decreasing the yield of full-length oligonucleotides.^2^ Various approaches based on joining multiple short oligonucleotides have been developed to overcome these limitations with the goal of assembling long synthetic DNA strands (genes and gene fragments).^3^–^5^ Currently two main strategies are used, both based on procedures that involve the use of enzymes. The first utilises DNA ligase whereas the second relies on the activity of DNA polymerases.^3^,^4^ In the ligation method, which represents the earliest example of synthetic gene synthesis, the double-stranded DNA is assembled from complementary overlapping strands subsequently joined by the ligase to produce longer fragments, which requires 5′-phosphorylated oligonucleotides. This method becomes inefficient when large numbers of oligonucleotides are ligated.^3^ In the second method, called DNA polymerase cycling assembly (PCA), which is based on the activity of DNA polymerase enzymes, the desired gene fragment is produced in a multiple step assembly.^4^ Although these methods give access to a large variety of DNA fragments, there are some limitations due to mispriming, formation of secondary structures, and mistakes that occur when assembling repetitive sequences that hinder the polymerase activity. Therefore, in the rapidly evolving genomic, epigenomic and DNA nanotechnology fields the demand for functional gene fragments are not yet fully met, and alternative approaches are urgently needed.

To address this issue non-enzymatic ligation methods have been explored. Brown and collaborators reported a biocompatible chemical linkage that is read by polymerases both *in vitro* and *in vivo* in *E. coli* and human cells.^5^–^9^ The authors previously proved the efficiency of copper-mediated azide/alkyne cycloaddition (CuAAC),^10^,^11^ the classic click chemistry reaction,^12^ as a ligation method for oligonucleotides. A major advantage of this technique is the high reaction efficiency in aqueous buffer, which makes CuAAC a suitable reaction for conjugation of biomolecules.^13^

Following this work, recently, Kukwikila *et al.* demonstrated enzyme-free, click-mediated gene assembly starting from 10 functionalized oligonucleotides that overlap to create a small 335 bp gene. The assembled gene is functional both *in vitro* and *in vivo*, confirming the biocompatibility of the triazole linkage.^14^ The assembly approach used by Kukwikila *et al.* is based on splint oligonucleotides, but it is known that when increasing the number of strands to create a long gene, the complexity of the assembly increases significantly, often leading to failure in the synthesis of full-length sequences.^15^ An alternative method to chemically ligate DNA strands is based on formation of phosphoramidate linkages. In this case, 3′-amino-modified oligonucleotides react with 5′-phosphorylated partner strands in templated reactions. This method has been recently used for gene synthesis^16^ and also to circularize DNA nanostructures.^17^ The field of DNA nanotechnology has produced a large number of sophisticated 2D and 3D DNA nanostructures that have been applied in many research fields. It has been proven that DNA origami is a robust assembly method where a long single-stranded DNA scaffold is folded with the help of short DNA strands called staples.^18^ DNA origami has been widely used for organization of bio/nanomaterials at nanoscale precision, however, the use of DNA origami for gene assembly has not been reported. One of the great features of this technique is that the designed structure has the most stable conformation among all possible.^19^ Therefore it provides control over position and stoichiometry of each strand involved in the assembly. In this manuscript we explore these features and adapt them to gene synthesis. For this purpose, we tested a derivative of the DNA origami technique, where the scaffold is fragmented into ∼60 nt long strands (gene oligonucleotides, GOs) and it folds with the help of staple strands.

In this manuscript we describe a one-pot click assembly procedure inspired by established self-assembly techniques from the DNA nanotechnology field, affirming that genes can be obtained by chemical ligation of several short DNA strands. The work paves the way to the synthesis of long DNA fragments and genes by combining the geometrical precision achieved with DNA nanostructures and the highly efficient click chemistry-mediated ligation. In this work we demonstrate the synthesis of a 762 bp gene encoding the enhanced green fluorescent protein (EGFP) from 14 functionalized oligonucleotides. The system employs a DNA nanostructure – a 6-helix bundle (6HB)^20^ – as vehicle for assembling single stranded DNA bearing triazole linkages that are converted into double strands after PCR amplification. Using this technique, we assembled a 6HB where all GOs (3′ alkyne, 5′ azide-modified) are brought in close proximity, ordered in a predesigned fashion with an equimolar stoichiometry and ligated through click chemistry. The resulting product is then amplified by PCR to convert the triazole linkages in a canonical phosphodiester backbone (Fig. 1). The design was executed using the caDNAno software package^21^ using the following principles: (1) the 762 nt long gene runs through the nanostructure forming the single-stranded scaffold of a 6HB of ∼40 nm in length. (2) The gene scaffold is fragmented into strands of ∼60 nt to assure reliable chemical synthesis of the double functionalized GOs. (3) The staples are designed to allow the structure to fold in a hierarchical order. The gene was divided into 14 GOs; 12 internal GOs bearing a 5′-terminal azide-modified thymidine and a 3′-terminal alkyne-modified deoxycytidine and two terminal GOs mono-functionalized as 5′-azide and 3′-alkyne respectively.

**Fig. 1:**
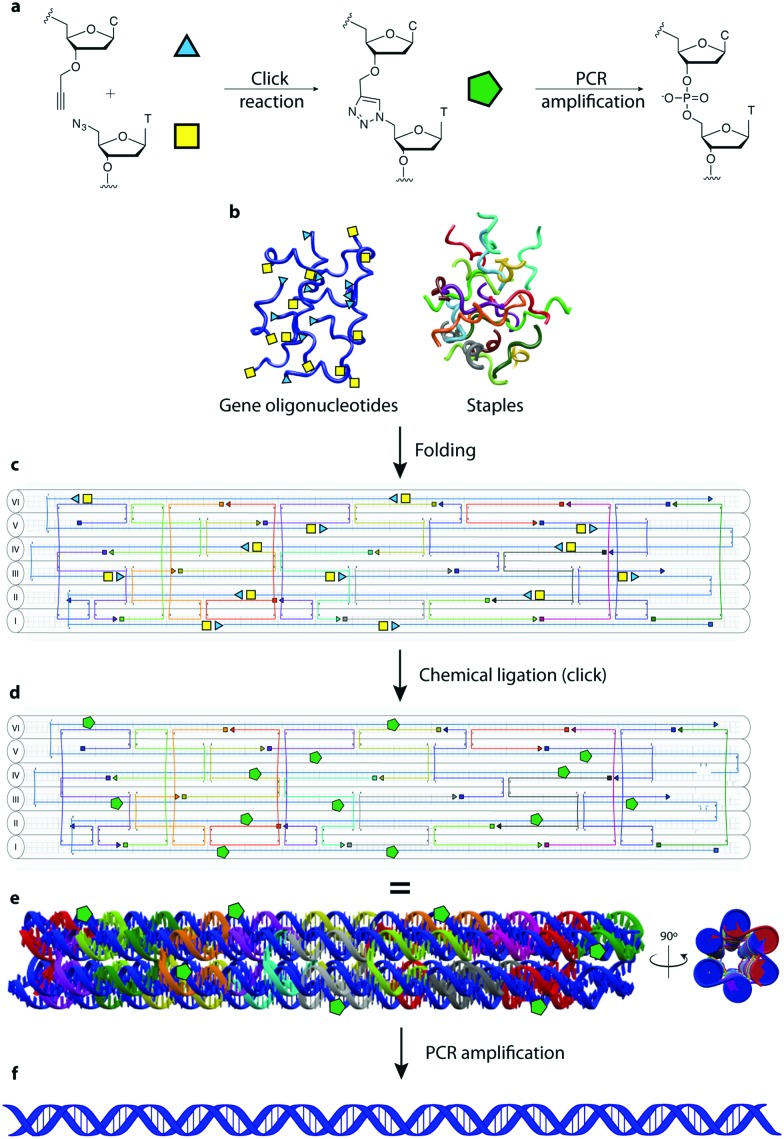
Gene assembly process. (a) Chemical ligation mechanism. Coloured shapes represent the molecule on their left. (b) Gene oligonucleotides (blue, GOs) – bearing chemical modifications at the 3′ and 5′-ends – and staples (colours other than blue) are folded, forming the 6HB construct (c) caDNAno blueprint: helices are marked with roman numerals. (d) Click chemistry of the GOs in the 6HB forms a long linear scaffold (in blue). (e) 3D views of the designed DNA nanostructure with green pentagons to highlight click points (triazoles). (f) The ligated DNA strand in the nanostructure is used as a template for PCR amplification forming a product with a canonical phosphate backbone. In figure c and d, the tips of the small arrows represent the 3′-end of DNA and the small squares represent the 5′-end.

To test the folding of the 6HB nanostructure, unmodified GOs were initially used. We found that the 6HB presented here folds in presence of 20 mM MgCl_2_ with formation of two species (Fig. 2a). The sample was analyzed by AFM and, as expected, the species were found to be monomers and dimers of the designed 6HB, with an average length of 43 ± 4.5 nm for the monomers and 82 ± 3.4 nm for the dimers (Fig. 2e). The length of monomers from AFM results agrees with theoretical calculation (42 nm). Dimer formation is probably due to stacking interactions between terminal base pairs of two different 6HB. The fact that only dimers, but not trimers or larger assemblies are formed, indicates that only one end of the 6HB tends to participate in base stacking. We speculated, and later confirmed, that the presence of the two species does not interfere with the gene assembly.

**Fig. 2:**
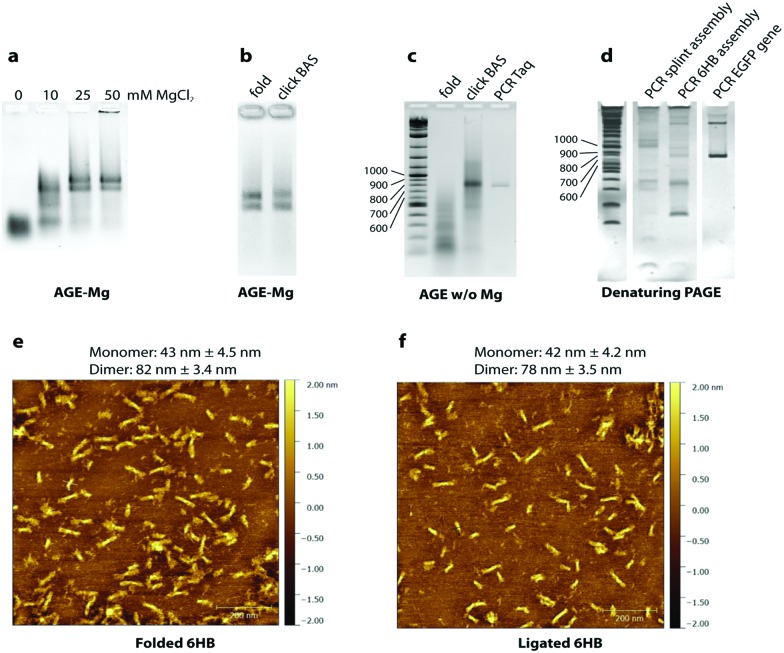
Assembly experiments. (a) Salt screening test (mM MgCl_2_): the structure formed at concentrations above 10 mM. Two species products (monomer and dimer) were found. (b) The product of the click reaction with the heterogeneous catalyst contained in the vial “reactor M” (BAS) runs like the folded sample. (c) The 6HB after click reaction is stable in absence of Mg ions. The PCR with *Taq* polymerase shows a product of the correct length. (d) Comparison between splint assembly without a nanostructure and assembly in the 6HB. The latter shows a product of the same length as the positive control (last lane, PCR on the EGFP gene). (e) AFM of the folded sample: monomers of ∼43 nm and dimers of ∼82 nm are formed. (f) AFM of the ligated sample: monomers of ∼42 nm and dimers of ∼78 nm are shown.

We then carried out chemical ligation of the EGFP gene, folding the 6HB using the 14 modified GOs and the complementary staples. Ligation through click chemistry – assisted by the close proximity of the GOs pre-organized in the nanostructure – was conducted using a protocol from Cassinelli *et al.*^22^ The gel containing magnesium ions (AGE-Mg) in Fig. 2b shows that the structure retains its conformation after the click reaction. In contrast, in an AGE without magnesium ions (Fig. 2c), the structure prior to the click reaction unfolds, whereas the ligated structure (“click BAS” in Fig. 2c) entirely retains its conformation. Chemically-ligated 6HB constructs were examined by AFM and they were shown to have a similar length to the 6HB having an unligated scaffold (42 ± 4.2 nm for the monomer and 78 ± 3.5 nm for the dimer) (Fig. 2f). However, PAGE analyses in denaturing conditions of the EGFP gene assembly was not very informative: many bands formed after the click reaction without any particular predominance (Fig. S1, ESI†). We speculated that the band corresponding to the full-length gene might be the closest to the well of the denaturing gel, but since we observed that triazole-containing oligonucleotides run slower than their unmodified counterpart, we could not reliably refer to the ladder in assessing their size.

The crude chemical ligation mixture was then used as template for PCR amplification. Primers were designed in order to amplify only the full length gene. Amplification of the full-length EGFP gene was successful when both *Taq* polymerase (low fidelity) and Baseclick polymerase (high fidelity) were employed (Fig. 2c). To assess the accuracy of the gene assembly method, PCR products were cloned and sequenced. In both cases, 5% of the screened clones resulted in 100% identity with the designed gene sequence (Tables S1–S3, ESI†). This is an encouraging result if we consider that one of the polymerase tested in the PCR step is *Taq* polymerase, classified as relatively low-fidelity due to its error-rate of 2.3 × 10^–5^ (*vs.* 9.5 × 10^–7^ of a high fidelity polymerase).^23^ At this point we calculated the error rate of the polymerase in our gene assembly method to understand if the system is prone to mutations, or whether the triazole groups interfere with the correct incorporation of bases during PCR. An estimation of the error rate of the system was obtained by comparing our results to published data for the fidelity of *Taq* polymerase, which is reported to incorporate 1 error every 700–1700 bp depending on the source of the mutation data.^24^ In our system *Taq* polymerase incorporated 1 error every 254 bp. This may indicate that the high concentration of metal ions present in the crude ligation mixture used as template for PCR may interfere with the activity of DNA polymerases, this can be addressed by exchanging the ion rich buffer with water. However, we cannot exclude that some of these mutations may be produced during the chemical synthesis of the starting oligonucleotides. Sequencing results show that mutations are homogeneously localized along the assembled gene fragment and that the triazole backbone is correctly replicated as previously shown.^5^

Finally, the method was compared to splint-assisted ligation in the absence of a nanoconstruct to prove the utility of the DNA nanostructure in assembling multiple gene oligonucleotides in equimolar ratio. The 14 GOs were assembled using 13 complementary splint oligonucleotides and chemically ligated with the same procedure used for the 6HB nanostructure. The ligation product was used as template for a PCR reaction where we employed KOD XL DNA polymerase, which is expected to efficiently read through the triazole linkage. Fig. 2d shows the PCR products of the splint-mediated assembly, the 6HB assembly and a positive control (PCR of the EGFP gene). PCR of the splint assembly did not produce full-length EGFP gene, but artifacts of higher and lower molecular weight, while PCR of the 6HB assembly showed a product of the same length as the control. However, the PCR products obtained using KOD XL polymerase were not as homogeneous as the ones employing *Taq* polymerase or Baseclick polymerase.

In conclusion, we have developed a system for gene fragment assembly by chemical ligation promoted by a DNA nanostructure, where gene fragments are part of the scaffold that runs inside the nanostructure. These are assembled in a predefined fashion, so that 3′-alkyne and 5′-azide are in close proximity, forming a 6HB nanostructure. The use of the nanostructure proved to be an efficient method to achieve an equimolar ratio of oligonucleotides, which is otherwise difficult when several oligonucleotides have to be ligated together. With this technique we were able to assemble 14 gene oligonucleotides to create a 762 nt long DNA strand, that after PCR is converted into a canonical double-stranded gene, encoding for EGFP. The method proved to be more efficient than the equivalent ligation performed using splint oligonucleotides in the absence of the nanostructure. The chemical ligation method based on the CuAAC reaction is fast and efficient and can be carried out in a variety of biologically compatible buffers. Interestingly this gene is twice the size of the only one previously synthesized by CuAAC-mediated ligation.^15^ This method should provide a general route to the synthesis of larger genes as well as long DNA strands for use in DNA nanotechnology and synthetic biology for the construction of complex nanostructures and synthetic organisms. We further envision the use of this technology to chemically assemble genes decorated with modifications such as epigenetic bases, fluorophores or haptens, which could have important applications in the fields of DNA nanotechnology and synthetic biology.

I. M. thanks the European School of DNA Nanotechnology (EScoDNA), a Marie Curie ITN under FP7 (Grant Number 317110) for financial support. S. C. thanks the European Union's Horizon 2020 framework program for research and innovation under grant agreement No. 642023 (ClickGene) for his PhD grant. A. K. thanks the Danish National Research Foundation (Grant Number DNRF81). Work carried out by T. B. and A. H. E.-S. was funded by the UK BBSRC sLoLa grant BB/J001694/2: extending the boundaries of nucleic acid chemistry.

## Conflicts of interest

The authors declare no conflicts of interest.

## Supplementary Material

Supplementary information

## References

[cit1] Caruthers M. H. (1987). Methods Enzymol..

[cit2] Kosuri S., Church G. M. (2014). Nat. Methods.

[cit3] Chalmers F., Curnow K. (2001). Biotechniques.

[cit4] Stemmer W. P. C., Crameri A., Kim D. H., Brennan T. M., Heyneker H. L. (1995). Gene.

[cit5] El-Sagheer A. H., Sanzone A. P., Gao R., Tavassoli A., Brown T. (2011). Proc. Natl. Acad. Sci. U. S. A..

[cit6] Birts C. N. (2014). Angew. Chem., Int. Ed..

[cit7] El-Sagheer A. H., Brown T. (2015). Q. Rev. Biophys..

[cit8] El-Sagheer A. H., Brown T. (2011). Chem. Commun..

[cit9] El-Sagheer A. H., Brown T. (2012). Acc. Chem. Res..

[cit10] Rostovtsev V. V., Green L. G., Fokin V. V., Sharpless K. B. (2002). Angew. Chem., Int. Ed..

[cit11] Tornøe C. W., Christensen C., Meldal M. (2002). J. Org. Chem..

[cit12] Kolb H. C., Finn M. G., Sharpless K. B. (2001). Angew. Chem., Int. Ed..

[cit13] Gierlich J., Burley G. a., Gramlich P. M. E., Hammond D. M., Carell T. (2006). Org. Lett..

[cit14] Kukwikila M., Gale N., El-Sagheer A. H., Brown T., Tavassoli A. (2017). Nat. Chem..

[cit15] Hughes R. A., Miklos A. E., Ellington A. D. (2011). Methods Enzymol..

[cit16] El-Sagheer A. H., Brown T. (2017). Chem. Commun..

[cit17] Kalinowski M. (2016). ChemBioChem.

[cit18] Rothemund P. W. K. (2006). Nature.

[cit19] Dunn K. E. (2015). Nature.

[cit20] Douglas S. M. (2009). Nucleic Acids Res..

[cit21] Douglas S. M. (2009). Nature.

[cit22] Cassinelli V. (2015). Angew. Chem., Int. Ed..

[cit23] Thermo Scientific. PCR fidelity calculator. https://www.thermofisher.com/dk/en/home/brands/thermo-scientific/molecular-biology/molecular-biology-learning-center/molecular-biology-resource-library/thermo-scientific-web-tools/pcr-fidelity-calculator.html.

[cit24] McInerney P., Adams P., Hadi M. Z. (2014). Mol. Biol. Int..

