# Supplementary material for: Gene assembly via one-pot chemical ligation of DNA promoted by DNA nanostructures
Source: Chem Commun (Camb). 2018 Apr 17;54(36):4529–32. doi: 10.1039/c8cc00738a (PMC5944424; doi:10.1039/c8cc00738a)
Supplement: Supplementary file 1 [file CC-054-C8CC00738A-s001.pdf]

Electronic Supplementary Material (ESI) for ChemComm.
This journal is © The Royal Society of Chemistry 2018

Supplementary Information

Gene Fragment Assembly via One-Pot Chemical Ligation of DNA Promoted by Nanostructures

Ilenia Manuguerra[#1], Stefano Croce[#2,5], Afaf H. El-Sagheer[3,4], Abhichart Krissanaprasit[1], Tom Brown[3], Kurt V. Gothelf[1] and Antonio Manetto*[6]

[1]Interdisciplinary Nanoscience Center (iNANO), Gustav Wieds Vej 14 and Department of Chemistry, Langelandsgade 140, Aarhus University, 8000 Aarhus (Denmark);
[2]Center for Integrated Protein science, Department of Chemistry, Ludwig-Maximilians-Universität (LMU), Butenandtstrasse 5-13, 81377 Munich (Germany);
[3]Department of Chemistry, University of Oxford, Chemistry Research Laboratory, 12 Mansfield Road, Oxford, OX1 3TA, (United Kingdom);
[4]Chemistry Branch, Department of Science and Mathematics, Faculty of Petroleum and Mining Engineering, Suez University, 43721 Suez (Egypt);
[5]baseclick GmbH, Floriansbogen 2-4, 82061 Neuried Munich (Germany);
[6]Metabion GmbH, Semmelweisstrasse 3, 82152 Planegg Munich (Germany).
[#]Both authors contributed equally to this work.
*E-mail: a.manetto@metabion.com

Experimental procedures

Design of the 6HB

The design of the 6HB was supported by caDNAno software. After introducing the sequence of the scaffold the staples were added automatically by the software and then manually modified to achieve a hierarchical folding.
In Supplementary Figure 3 a high-resolution blueprint with gene oligonucleotides and staples is shown.

Folding of the 6HB

Samples were made mixing staples and in-house synthetized GOs in ratio 1:1 to a final concentration of 500 nM/each oligo in 1X TE buffer with 20 mM $MgCl_2$. The sample was folded in a thermocycler using the following program: from 95 ºC to 80 ºC with a ramp of 1º C/min, from 80 ºC to 40 ºC with a ramp of 0.03ºC/min, from 40 ºC to 23 ºC with a ramp of 0.1 ºC/min and final hold at 8 ºC.

Click reactions on the 6HB

- **Click reaction (CuAAC) using an heterogeneous catalyst**

A volume of 15 µl of THPTA 0.1 M was added to the “reactor M”, a vial containing the heterogeneous catalyst (baseclick), then 20 µl of folding reaction was added to the “reactor”. The sample was incubated at 32ºC with gentle shaking (200 rpm) for 5h.

- **Click reaction (CuAAC) using $CuSO_4$**

The Baseclick EdU kit (reaction buffer, catalyst solution and reducing agent/buffer additive) was used for the experiments with $CuSO_4$ as source of Cu(I). The indications of the producer were used for the ligation assay using the click reaction. In this case, 40 µl of folding reaction were used in the assays.

- **Click reaction (CuAAC) using CuBr**

For the experiments with CuBr as source of Cu(I): 1 mg of CuBr was weighted under inert atmosphere and dissolved in 70 µl of “click solution”. A total of 5 mg of the ligand TBTA were dissolved in 94 µl click solution and the two solutions were combined in ratio 1:2 (TBTA:CuBr). A volume of 20 µl of the mix were mixed with 4 µl of the folding reaction and incubated at 32ºC with gentle shaking.

AGE/AGE-Mg

AGE/AGE-Mg were prepared dissolving agarose (Ultra-pure, Thermo Scientific) to achieve a 1% gel in 0.5X TBE buffer (and 11 mM $MgCl_2$ final concentration for AGE-Mg, 1X TAE was used when the bands were extracted from gel). The gel was casted and left solidify at RT for 30 min.

PCR reactions

All the PCR reactions were tested using the following primers (Metabion):

FW EGFP 1-14	TATCACTATCGACGGTA
REV EGFP1-14	ACTTACAGCTTTACTTG

A volume of 1 µl of click reaction was used as template for PCR. The incubation with Taq Polymerase (NEB) and KOD XL (Millipore) proceeded as follows: 94 ºC for 3 min, 80ºC for 30 sec (add polymerase); 94 ºC for 45 sec, 30 ºC for 30 sec, 72 ºC for 12 min, repeat for 4 times; 94 ºC for 45 sec, 46 ºC for 30 sec, 72 ºC for 72 sec, repeat 10 times; 72 ºC for 10 min. Incubation with Baseclick polymerase proceeded as follows: 98 ºC for 90 sec; 98 ºC for 10 sec, 58 ºC for 20 sec, 72 ºC for 15 sec, repeat 20 times; 72 ºC for 8 min.

Sequencing

PCR reactions were cloned into a plasmid using the TOPO PCR cloning kit by Thermo Fisher Scientific and following manufacturer instructions. Plasmids from clones were extracted with Gene Elute HP Miniprep by Sigma-Aldrich and sent to sequencing to GATC Biotech.

AFM imaging

A volume of 2 µl sample was deposited on freshly cleaved mica and incubated for 1 min. Subsequently, 400 µl of imaging buffer (1× TAE-Mg2+ containing 5 mM NiCl2, 1× TAE-Mg-Ni) were added into the cell. Nanostructures were visualized by tapping mode AFM (Agilent AFM series 5100) using silicon nitride cantilevers (Olympus). All recorded AFM images were processed and analyzed by Gwyddion software.

Supplementary Figures

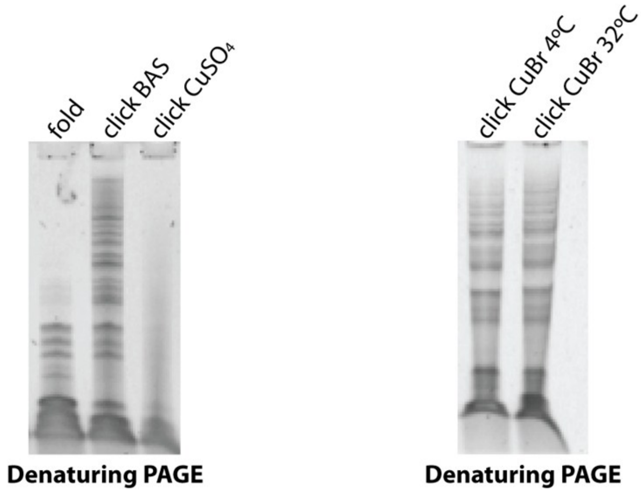

Supplementary Figure 1. Denaturing PAGE of the 6HB after folding and after ligation with different catalysts. Many bands are formed, but the complete EGFP gene is not detectable using PAGE, probably due to the low yield of the long sequence. To understand whether a different catalyst could give higher yields in the formation of the “ligated gene”, CuAAC reaction using $CuSO_4$ as source of Cu(I) was tested. The sample appears like a light smear, probably because the reagent interferes with either the electrophoresis or the staining of DNA. CuBr was also tested as source of Cu(I), but the results are similar to the ones obtained with the heterogeneous catalyst.

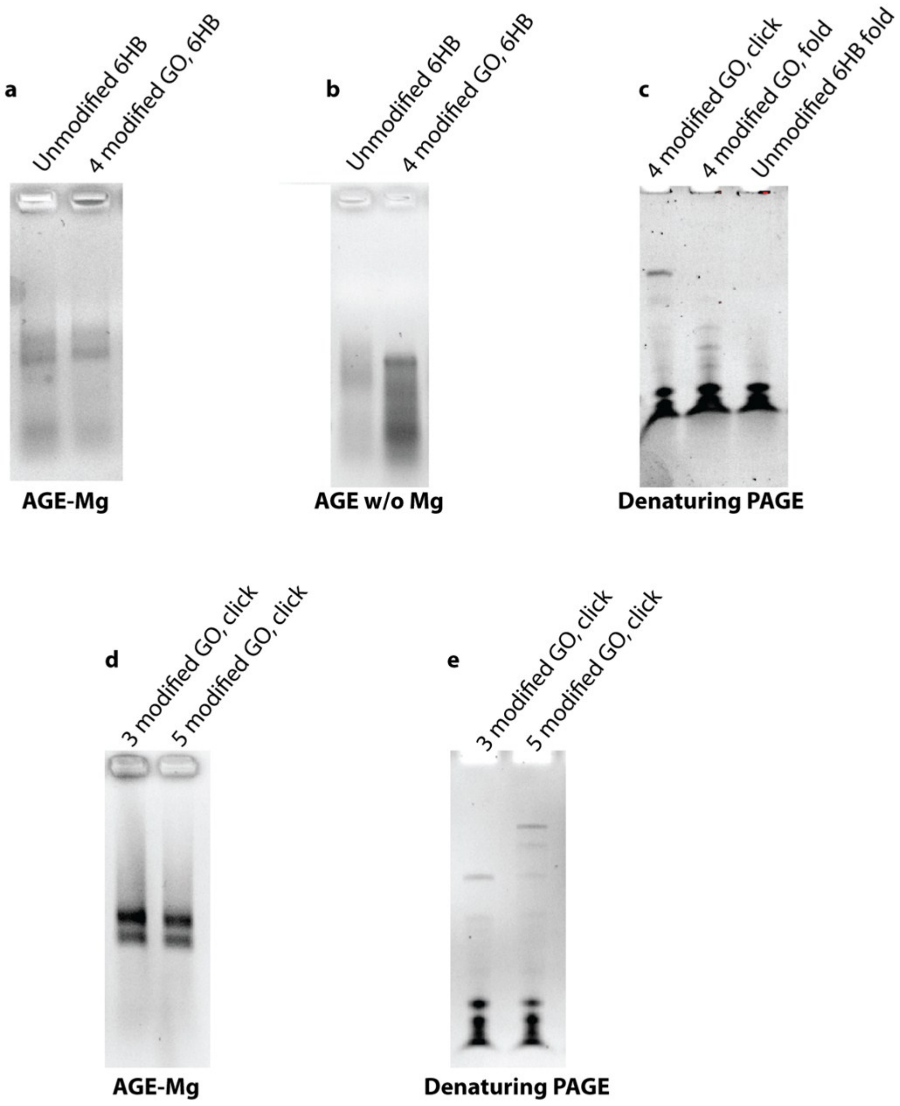

Supplementary Figure 2. Gene assembly in the 6HB of (a, b, c) 4, (d, e) 3 and 5 modified gene oligonucleotides. (a) Folding and click reaction on the 6HB using 4 modified gene oligonucleotides. The nanostructure forms using both a set of unmodified gene oligonucleotides or a set containing only 4 modified gene oligonucleotides. (b) Only the ligated structure partially retains its conformation in an AGE without Mg^{2+} ions. (c) Denaturing PAGE of the folded and ligated structures with 4 modified gene oligonucleotides or fully unmodified. Only after the click reaction is a band of high molecular weight corresponding to 4 ligated fragments visible (first lane). (d) Folding and click reaction on the 6HB using 3 and 5 modified gene oligonucleotides. (e) Denaturing PAGE of the ligation product in (d). Bands corresponding to 3 and 5 ligated gene oligonucleotides are visible on gel.

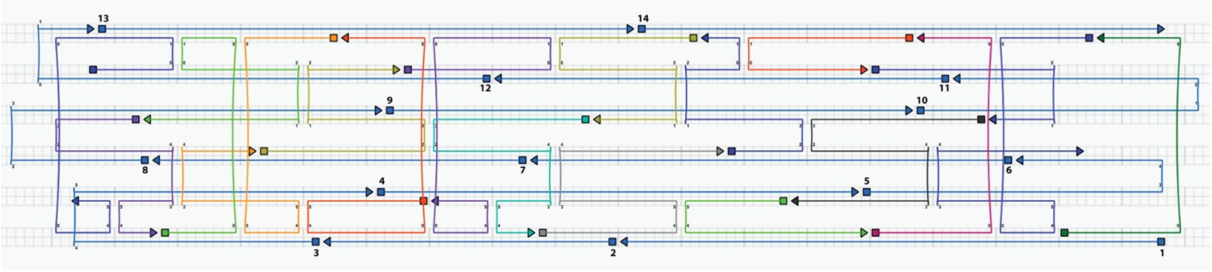

Supplementary Figure 3. Blueprint of the 6HB used in this manuscript. GOs are in blue, staples are represented in other colors. Arrow heads indicate the 3'end. Numbers on the 5'-end of the blue strands indicate the name of the GOs (i.e. 1 = EGFP1, 12 = EGFP12).

Supplementary Text

Sequencing result analysis of PCR products using Taq polymerase (medium-low fidelity)

Table S1: number of mutations (divided into substitutions, deletions and insertions) for each sequenced clone containing ~762 bp insert.

Clone Name	Substitutions	Deletions	Insertions
A02	2 (9 bp after click point)	1 (13 bp before click point)	1
B01	2 (12 bp, 9bp before click point)	1 (9 bp before click point)	1 (8 bp before click point)
D02	3 (16 bp before click point)	0	0
**D03**	**0**	**0**	**0**
H03	2 (13 bp before click point)	0	0
H04	1	0	0
**H06**	**0**	**0**	**0**

48 clones were sent for sequencing.

38 clones produced sequencing result.

33 clones had the PCR products as insert.

7 clones have a 762 bp insert.

2 clones (D03 and H06) have 100% identity with the designed sequence.

~5% of the clones that produced sequencing results showed 100% identity with the designed sequence.

Sequencing result analysis of PCR products using Baseclick polymerase (high fidelity)

Table S2: number of mutations (divided into substitutions, deletions and insertions) for each sequenced clone containing ~762 bp insert.

20 clones were sequenced

Clone name	Deletions	Substitutions	Insertions
EGFP_3_1	0	0	0
EGFP_3_2	3	1	0
EGFP_3_3	0	1	0
EGFP_3_4	0	2 + 2 c	1
EGFP_3_5	0	1	0
EGFP_3_6	0	1	0
EGFP_5_1	1	2	0
EGFP_5_2	2 c, 15 c (part of GO5)	2	0
EGFP_5_3	1	1	0
EGFP_5_4	60 c (whole GO10)+ 1	0	0
EGFP_5_5	0	2	0
EGFP_5_6	2 c	1	0
EGFP_5_7	3	0	0
EGFP_8_1	1 + 3 c	0	1
EGFP_8_2	1	0	0
EGFP_8_3	4	1	0
EGFP_8_4	0	1	0
EGFP_8_5	4	1	0
EGFP_8_6	2	2	0
EGFP_8_7	7	0	0
Tot (considering long deletions as 1)	37	21	2

(GO10: gene oligonucleotides EGFP10; 2 c: 2 consecutive mutations)

Table S3: Description of mutations close to click points

Clone name	Sequence surrounding CT click point (-: deletions)	Gene oligonucleotides involved
EGFP_5_2	C-CCTACG	3-4
	-CGCTACC	5-6
EGFP_5_4	GGAGTAC(-15 bp-)AACTTCAAGATCC	9-11 (10 is missing)
EGFP_5_5	CGACTACAACA	9-10
EGFP_5_6	C--CTGGAGTT	13-14
EGFP_3_2	CAACTAC-ACA	9-10
	TA-CTGAGCA	12-13

The click region of gene oligonucleotides 9-10 appears to be prone to mutations. We speculate that GO10 might not have enough complementary bases at the level of the staples (see Supplementary Figure 3). This might explain why gene oligonucleotides 10 (represented in red box, Fig Sx) is missing in one of the clones sequenced.

Oligonucleotides

All reagents for chemical synthesis were purchased from Sigma-Aldrich. Unmodified oligonucleotides were acquired from Metabion International AG, while 3'-alkyne and 5'-azide modified gene oligonucleotides were synthesized in-house using a previously reported procedure.[6]

Sequences

GENE OLIGONUCLEOTIDES (GOs)		
Name	Sequence (5' to 3' end)	Length (nt)
EGFP1	TCGACGGTACCGCGGGCCCGGGATCCACCGGTCGCCACCATGGTGAGCAAGGGCGAGGAGC	61
EGFP2	TGTTCACCGGGGTGGTGCCCATCCTGGTCGAGC	33
EGFP3	TGGACGGCGACGTAAACGGCCACAAGTTCAGCGTGTCCGGCGAGGGCGAGGGCGATGCCACC	62
EGFP4	TACGGCAAGCTGACCCTGAAGTTCATCTGCACCACCGGCAAGCTGCCCGTGCCC	54

EGFP5	TGGCCCACCCTCGTGACCACCCTGACCTACGGTGTACAGT GCTTCAGCCGC	51
EGFP6	TACCCCGACCACATGAAGCAGCACGACTTCTTCAAGTCCG CCATGCCCGAAGGC	54
EGFP7	TACGTCCAGGAGCGCACCATCTTCTTCAAGGACGACGGCA AC	42
EGFP8	TACAAGACCCGCGCCGAGGTGAAGTTCGAGGGCGACACC CTGGTGAACCGCATCGAGC	58
EGFP9	TGAAGGGCATCGACTTCAAGGAGGACGGCAACATCCTGGG GCACAAGCTGGAGTACAAC	59
EGFP10	TACAACAGCCACAACGTCTATATCATGGCCGACAAGCAGAA GAACGGCATCAAGGTGAAC	60
EGFP11	TTCAAGATCCGCCACAACATCGAGGACGGCAGCGTGCAGC TCGCCGACCAC	51
EGFP12	TACCAGCAGAACACCCCCATCGGCGACGGCCCCGTGCTGC TGCCCGACAACCACTACC	58
EGFP13	TGAGCACCCAGTCCGCCCTGAGCAAAGACCCCAACGAGAA GCGCGATCACATGGTCCTGC	60
EGFP14	TGGAGTTCGTGACCGCCGCCGGGATCACTCTCGGCATGGA CGAGCTGTACAAGTAAAGC	59
**STAPLE STRANDS**		
Name	Sequence (5’ to 3’ end)	Length (nt)
I6M0	GCCGTAGGTGGCATCAGCTCGACCAGGATCGTTGGGGT	38
I6M1	AAGAAGTCGCTTGTGCCCCAGGAGCCGTCCTCACG	35
I6M2	CTCCTTGAAGTCGATGCCTCCTGGACGTAGCCTTCAGGGC ACCC	44
I6M3	GGGCAGCAGCGGGTGCTCAGGTAGTTAACTTCGCTG	36
I6M4	GCAGCTTGCCGGGCCCTTGCTCACCATGGTGGC	33
I6M5	GATCTTGAAGTTCACCTTGATCGTTGTGG	29
I6M6	AACTCCAGCAGGACCAGCGAGCTGCACGCTTGTTGCCGTC	40
I6M7	CGGTGAACAGCTCCTCTGGTGCAGATGAACTTCGGGCATG GCGGACTTG	49
I6M8	CTTTGCTCAGGCGCCGTCCGCCCTCGCCCTCGGCCGTCGT C	41
I6M9	CTGTTGTAGTTGTACTCCAGTGCTGCTTCATGTGGTGGGCC AGGGCACGG	50
I6M10	GACCGGTGGATCCCTCCATGCCG	23
I6M11	TGTACAGCTCGGGGCCCGGGGTGGTCACGAGGGTCGGGG TAGCGGCTGA	49
I6M12	CGTTTACGTGCGGACTACGGGGCCGTCGCCGGTTCACCAG GGTGTCGC	48
I6M13	CCTCGAACTTCGGGTCTTGTAGTTCCGGACAGTGGC	36
I6M14	TCTGCTGGTAGTGGTCGTGTGATCGCGCTTCTGGGCACTC	45

	AGCTT	
I6M15	AGAGTGATCCCGGCGGCGGTCGATGTTGTGGCG	33
I6M16	CGGTACCGTCGATTTTGCTTTACT	24
I6M17	CTTGAAGAAGATGGTGCGCCTTCAGCTCGATGCGATGGGG GTGT	44

EGFPn modified sequences were synthesized with 3'-alkyne and 5'-azide.

Typical LC/MS analyses are shown in the following chromatograms and deconvoluted mass spectra.

LC/MS EGFP6

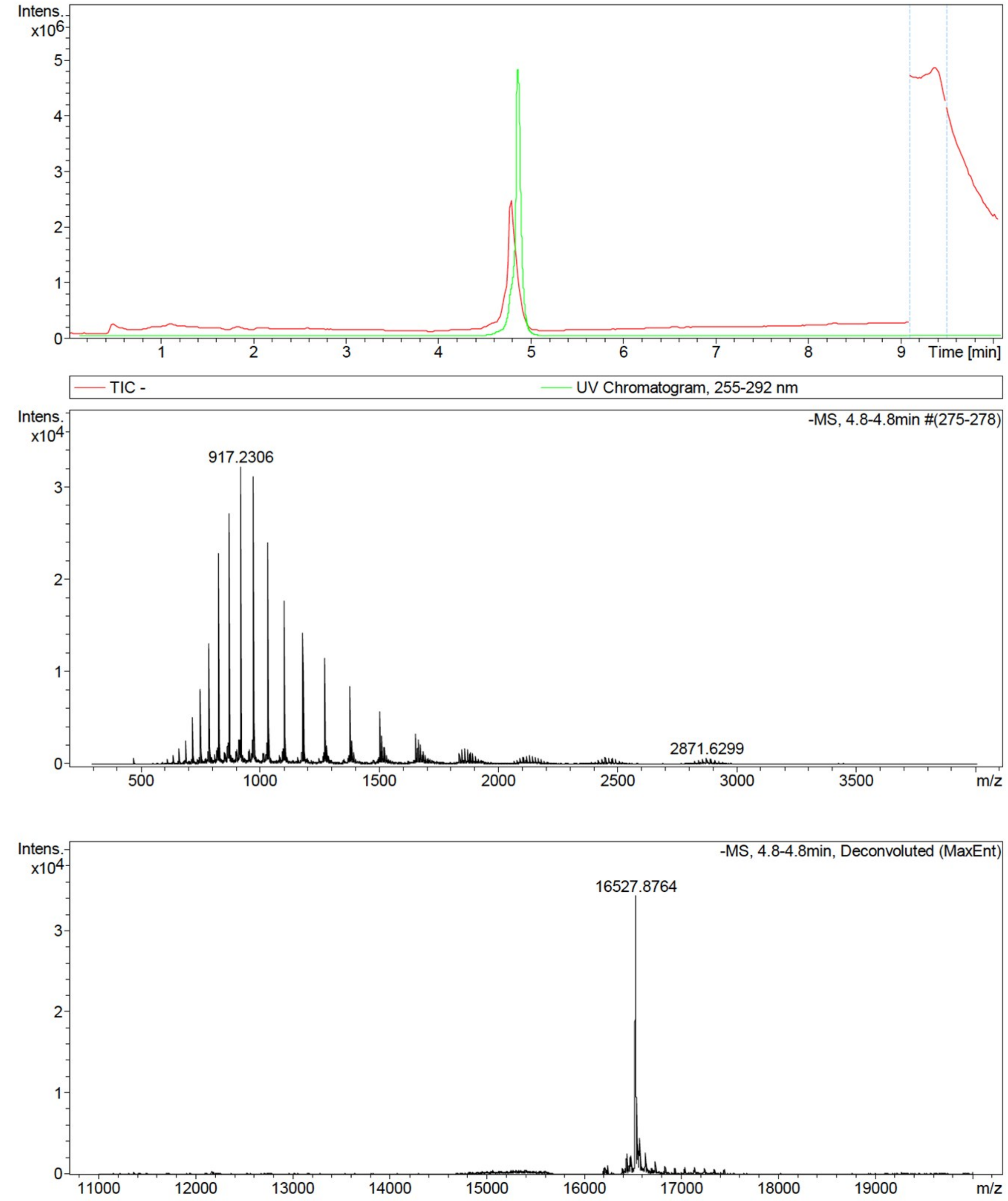

Intens.
x10^6
Time [min]
TIC -
UV Chromatogram, 255-292 nm
Intens.
x10^4
-MS, 4.8-4.8min #(275-278)
917.2306
2871.6299
m/z
Intens.
x10^4
-MS, 4.8-4.8min, Deconvoluted (MaxEnt)
16527.8764
m/z

LC/MS EGFP8

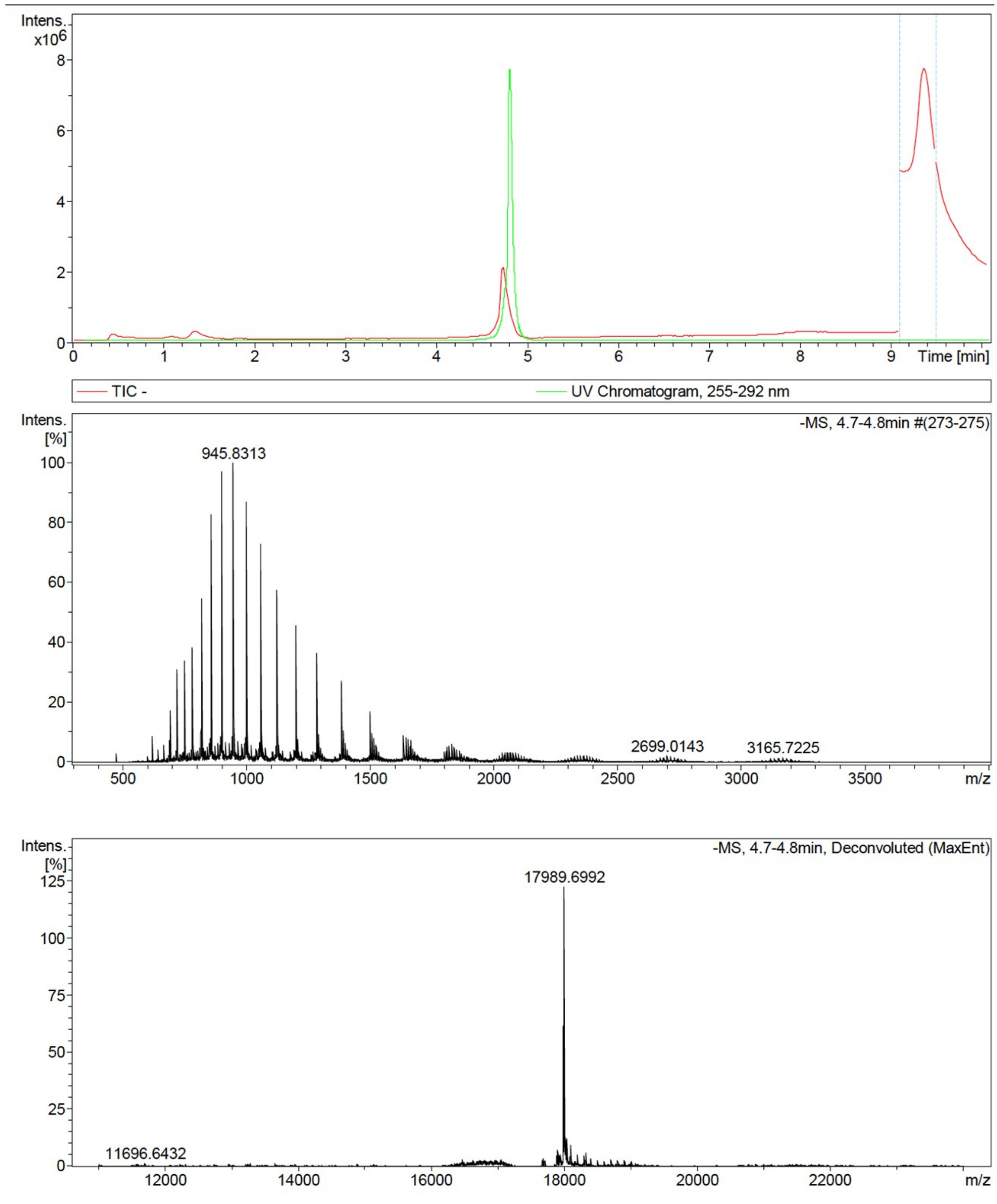

Intens.
x10^6
8
6
4
2
0
0 1 2 3 4 5 6 7 8 9 Time [min]
TIC -
UV Chromatogram, 255-292 nm
Intens.
[%]
-MS, 4.7-4.8min #(273-275)
945.8313
100
80
60
40
20
0
2699.0143
3165.7225
500 1000 1500 2000 2500 3000 3500 m/z
Intens.
[%]
-MS, 4.7-4.8min, Deconvoluted (MaxEnt)
17989.6992
125
100
75
50
25
0
11696.6432
12000 14000 16000 18000 20000 22000 m/z

LC/MS EGFP12

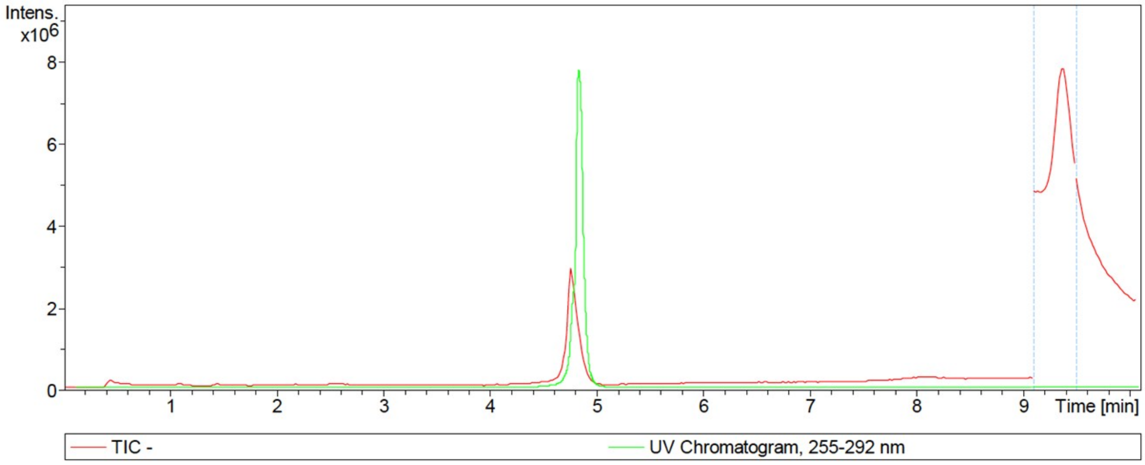

Intens.
x10^6
8
6
4
2
0
1
2
3
4
5
6
7
8
9
Time [min]
TIC -
UV Chromatogram, 255-292 nm

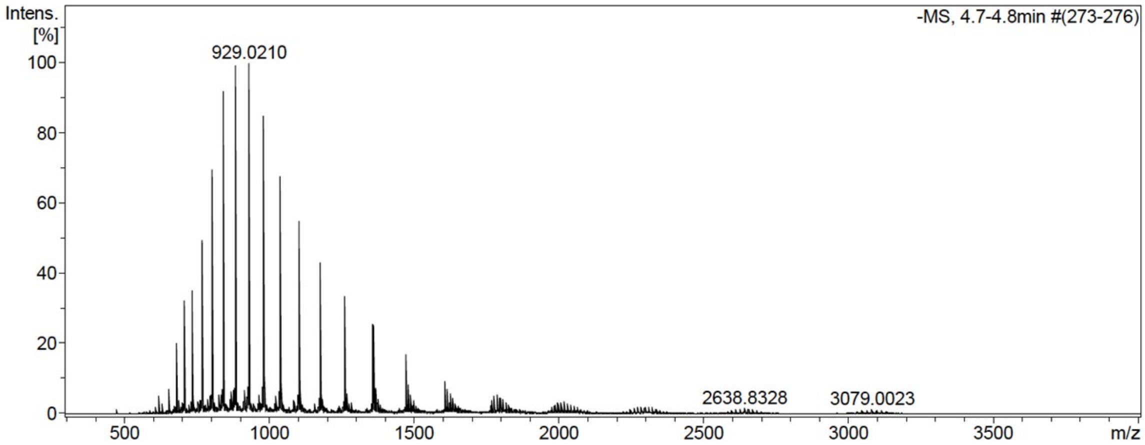

Intens.
[%]
-MS, 4.7-4.8min #(273-276)
929.0210
2638.8328
3079.0023
100
80
60
40
20
0
500
1000
1500
2000
2500
3000
3500
m/z

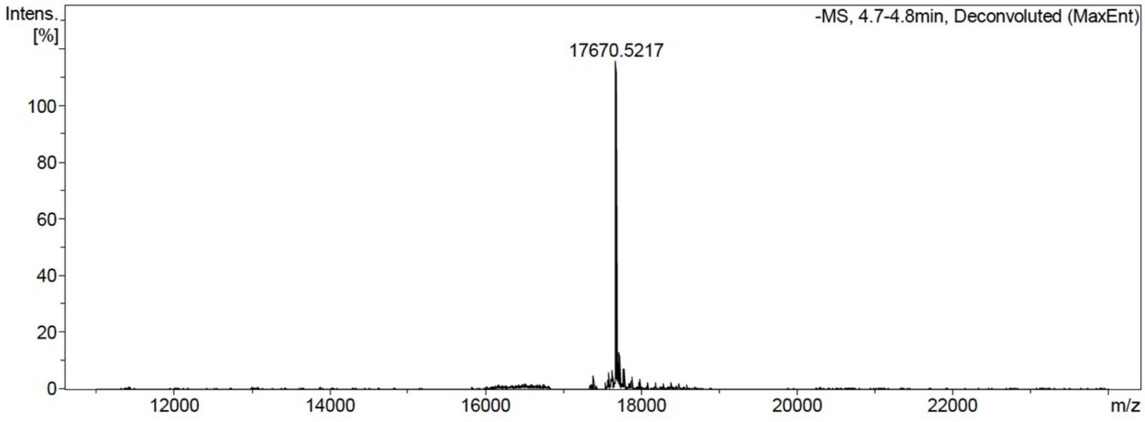

Intens.
[%]
-MS, 4.7-4.8min, Deconvoluted (MaxEnt)
17670.5217
100
80
60
40
20
0
12000
14000
16000
18000
20000
22000
m/z
